# Atypical fibroxanthoma in sun-protected sites: two cases in the inguinal region and thigh^؆^

**DOI:** 10.1016/j.abd.2026.501337

**Published:** 2026-04-18

**Authors:** Ho Sung Kim, Won Gyu Choi, Song Hee Oh, Soo-Kyung Lee, Un Ha Lee, Myoung Shin Kim

**Affiliations:** Department of Dermatology, College of Medicine, Sanggye Paik Hospital, Inje University, Seoul, South Korea

Dear Editor,

Atypical fibroxanthoma (AFX) is an uncommon cutaneous soft-tissue neoplasm with low metastatic potential but occasional local recurrence.^1^, ^2^ It typically arises on sun-exposed head and neck skin in older patients, while cases on sun-protected regions are uncommon.^3^, ^4^, ^5^ We report two cases of AFX arising in the inguinal region and thigh of middle-aged immunocompetent men.

## Case 1

**Fig. 1:**
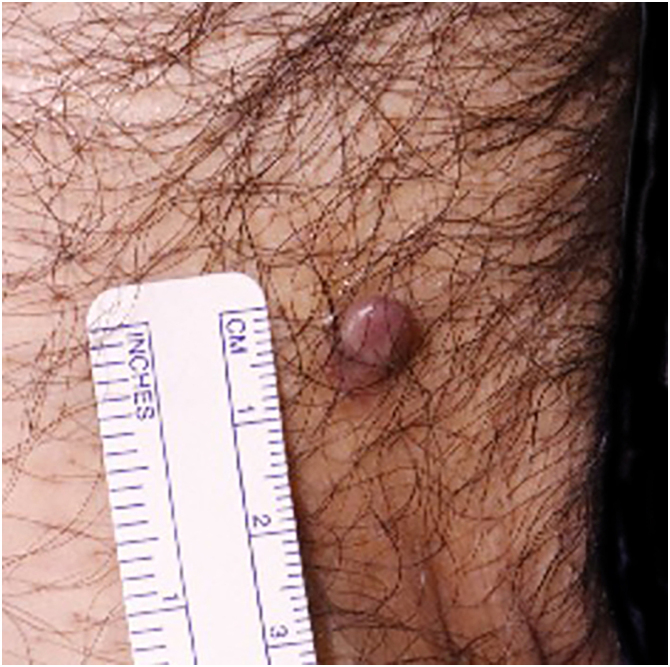
Case 1: Atypical fibroxanthoma on the inguinal region.

**Fig. 2:**
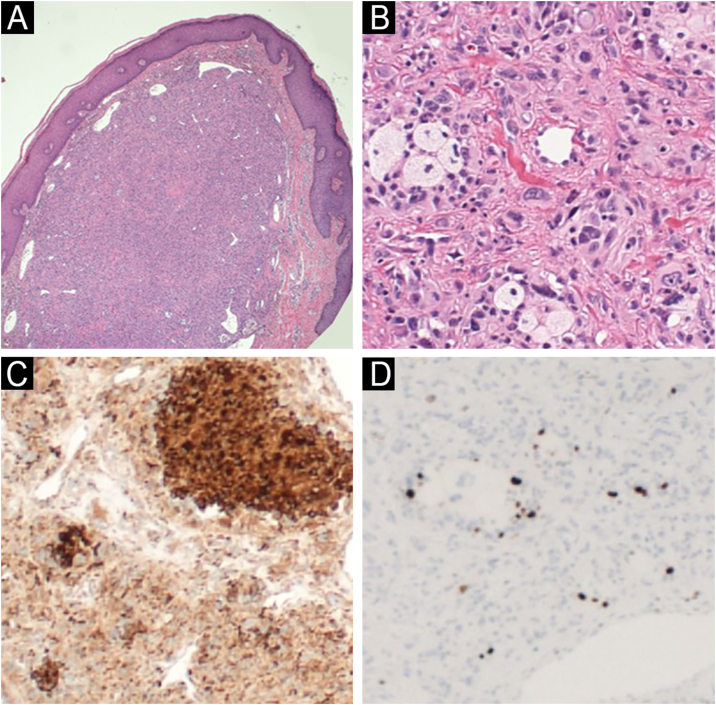
Case 1: (A) Hematoxylin and eosin staining showing a well-demarcated, non-encapsulated dermal tumor (40× magnification). (B) Hematoxylin & eosin staining showing pleomorphic spindle and epithelioid cells, as well as foamy histiocyte-like cells with atypical mitoses (400× magnification). (C) CD68 Immunohistochemistry (IHC) showing strong positivity in the tumor cells (100× magnification). (D) Ki-67 IHC showing a labeling index of around 10% in tumor cells (100× magnification).

A 54-year-old man presented with a 7–8 mm smooth, grayish-brown papule on the inguinal region that had persisted for 7–8 months (Fig. 1). The patient had no history of trauma, radiotherapy, or immunosuppression. Histology showed a well-demarcated dermal tumor without subcutaneous invasion, with pleomorphic spindle and epithelioid cells, foamy histiocyte-like cells, and atypical mitoses (Fig. 2 A‒B). Immunohistochemistry (IHC) revealed CD68 positivity, partial CD31 positivity, and negativity for CD34, HMB45, and pan-cytokeratin, with Ki-67 ∼10% (Fig. 2C‒D). The lesion was completely excised, and no metastasis was detected.

## Case 2

**Fig. 3:**
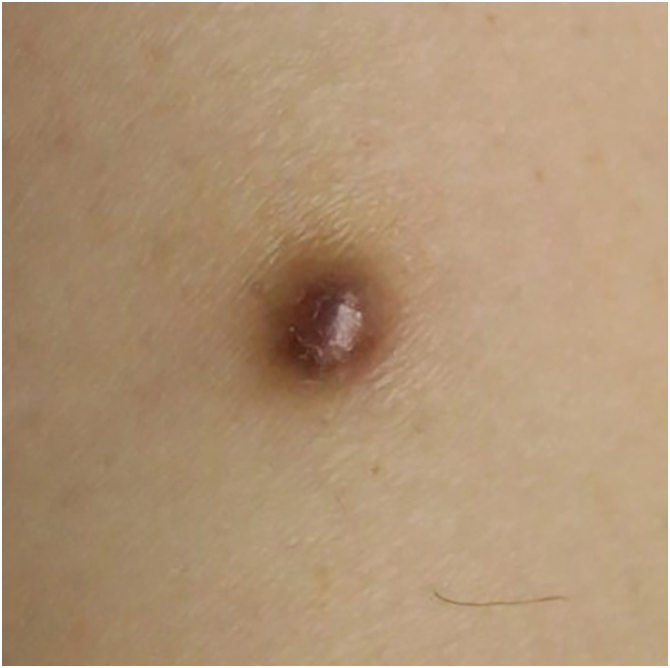
Case 2: Atypical fibroxanthoma of the right thigh.

**Fig. 4:**
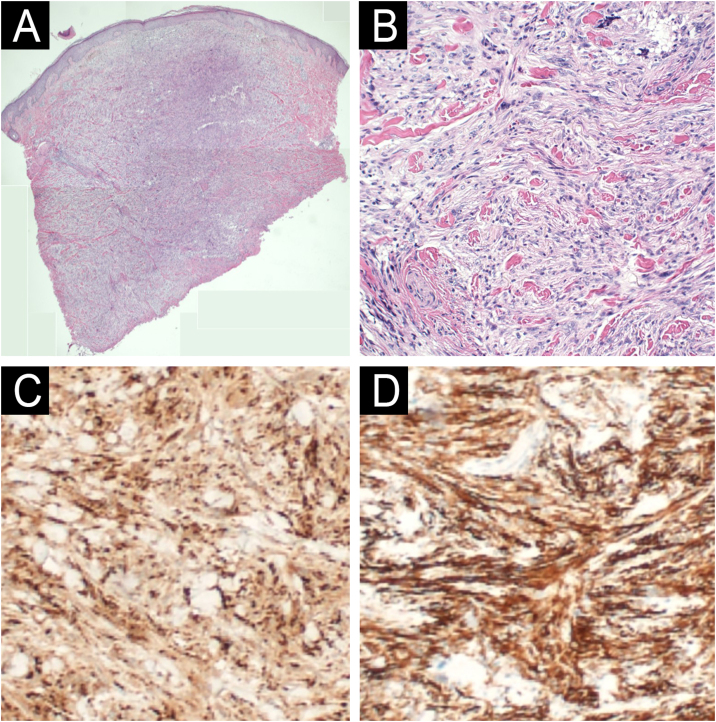
Case 2: (A) Hematoxylin & eosin staining showing a dermal-based spindle cell tumor (40× magnification). (B) Hematoxylin & eosin showing spindle cells with pleomorphic nuclei and foamy cells (200× magnification). (C) CD68 IHC showing strong positivity in the tumor cells (100× magnification). (D) SMA IHC showing strong positivity in the tumor cells (100× magnification).

A 53-year-old man presented with a 6 mm smooth, dark-brown papule on the right thigh that had persisted for 5-years (Fig. 3). He was a hepatitis B virus carrier but otherwise immunocompetent. Histology revealed a dermal tumor of densely packed spindle cells with pleomorphic nuclei and foamy cells (Fig. 4A‒B). IHC revealed CD68, CD10, and SMA positivity, CD34 negativity, and Ki-67 ∼10% (Fig. 4 C‒D). The patient was diagnosed with spindle-cell-type AFX. Re-excision was recommended, but the patient declined; a two-month follow-up revealed no recurrence.

AFX typically presents as a solitary dome-shaped papule or nodule on sun-exposed skin in older males. Ultraviolet (UV) radiation is regarded as the main etiological factor, inducing p53 mutations and UV-specific DNA damage.^2^ Other contributing factors include previous radiation exposure, trauma, or immunosuppression.^1^

AFX on sun-protected sites is exceptionally uncommon, with few cases reported on the trunk, proximal limbs, or genital regions.^3^, ^4^, ^5^ Koch et al.^1^ noted that only ∼15% of AFX cases involved the trunk or extremities, with even fewer arising in the thigh or inguinal region. Our patients were relatively young, immunocompetent, and lacked known risk factors, suggesting that other mechanisms may be involved in the condition’s etiology. Recent genomic studies have identified that recurrent alterations in FAT1, NOTCH1/2, CDKN2A, TP53, and the promoter of the *TERT* gene may be linked to AFX.^6^ Some AFX-associated mutations are clearly UV-related (e.g., C>T transitions), whereas others (e.g., locus loss or copy number variations) can arise either from UV-induced genomic instability or through UV-independent mechanisms, highlighting alternative pathogenetic pathways.^6^

Histologically, AFX is a well-circumscribed, dermal-based tumor that spares subcutaneous and adnexal structures. Pleomorphic spindle and epithelioid cells, foamy histiocyte-like cells, and atypical mitoses are characteristic.^1^, ^2^ Differential diagnoses include Pleomorphic Dermal Sarcoma (PDS), Undifferentiated Pleomorphic Sarcoma (UPS), melanoma, leiomyosarcoma, poorly-differentiated squamous cell carcinoma, Atypical Dermal Fibroma (ADF), Malignant Dermal Fibroma (MDF), and Dermatofibrosarcoma Protuberans (DFSP). PDS typically shows deeper invasion and perineural or lymphovascular involvement, whereas UPS is more aggressive and frequently metastasizes.^7^ ADF and MDF can be differentiated from AFX, as ADF shows a well-defined grenz zone, epidermal hyperplasia, and fewer atypical mitoses (<1%), while MDF exhibits deep invasion and necrosis, with repeated local recurrences.^8^, ^9^ IHC is essential, as AFX typically expresses CD68 and vimentin, but is negative for cytokeratins, Factor XIIIa, and CD34. SMA or CD10 positivity may also be present in spindle-cell variants.^1^ By contrast, SCC is cytokeratin-positive, melanoma expresses SOX-10 or Melan-A, leiomyosarcoma expresses SMA/desmin, ADF and MDF express Factor XIIIa, and DFSP shows strong CD34 positivity.^1^, ^8^, ^9^

AFX has a favorable prognosis with low metastatic potential but ∼5% recurrence.^1^, ^2^ Complete surgical excision with negative margins remains the standard treatment, and Mohs micrographic surgery offers the lowest recurrence (∼2% vs. ∼9% for wide local excision).^10^ In Case 2, although the tumor involved all resection margins, no recurrence was observed during the two-month follow-up. Given the relatively short duration of follow-up, the possibility of local recurrence cannot be completely excluded, representing a limitation.

These two cases illustrate that AFX can arise in atypical, sun-protected sites, such as the inguinal region and thigh, even in middle-aged immunocompetent patients. Dermatologists and pathologists should remain aware of this possibility, as careful histopathological evaluation combined with IHC is crucial for accurate diagnosis and appropriate surgical management.

## ORCID ID

Ho Sung Kim: 0000-0001-5027-6871

Won Gyu Choi: 0009-0007-5888-1962

Song Hee Oh: 0009-0000-8747-9576

Soo-Kyung Lee: 0000-0002-7460-5657

Un Ha Lee: 0000-0003-1626-5583

Myoung Shin Kim: 0000-0002-0660-8098

## Financial support

None declared.

## Authors' contributions

Ho Sung Kim: Data collection, analysis and interpretation; critical literature review; preparation and writing of the manuscript.

Won Gyu Choi: Data collection, analysis and interpretation.

Song Hee Oh: Data collection, analysis and interpretation.

Soo-Kyung Lee: Critical literature review; intellectual participation in propaedeutic and/or therapeutic management of studied cases.

Un Ha Lee: Critical literature review; manuscript critical review; intellectual participation in propaedeutic and/or therapeutic management of studied cases.

Myoung Shin Kim: Study conception and planning; effective participation in research orientation; intellectual participation in propaedeutic and/or therapeutic management of studied cases; approval of the final version of the manuscript.

## Research data availability

Does not apply.

## Conflicts of interest

None declared.
